# Alantolactone promotes ER stress‐mediated apoptosis by inhibition of TrxR1 in triple‐negative breast cancer cell lines and in a mouse model

**DOI:** 10.1111/jcmm.14139

**Published:** 2019-01-04

**Authors:** Changtian Yin, Xuanxuan Dai, Xiangjie Huang, Wangyu Zhu, Xi Chen, Qiulin Zhou, Canwei Wang, Chengguang Zhao, Peng Zou, Guang Liang, Vinothkumar Rajamanickam, Ouchen Wang, Xiaohua Zhang, Ri Cui

**Affiliations:** ^1^ Chemical Biology Research Center School of Pharmaceutical Sciences, Wenzhou Medical University Wenzhou Zhejiang China; ^2^ Department of Thyroid and Breast Surgery The First Affiliated Hospital of Wenzhou Medical University Wenzhou Zhejiang China; ^3^ Cell and Molecular Biology Laboratory Zhoushan Hospital of Wenzhou Medical University Zhoushan Zhejiang China; ^4^ Affiliated Yueqing Hospital and School of Pharmaceutical Sciences, Wenzhou Medical University Wenzhou Zhejiang China

**Keywords:** alantolactone, ER‐stress, ROS, Triple‐negative breast cancer, TrxR1

## Abstract

Triple‐negative breast cancer (TNBC) is a subtype of breast cancer with poor clinical outcome and currently no effective targeted therapies are available. Alantolactone (ATL), a sesquiterpene lactone, has been shown to have potential anti‐tumour activity against various cancer cells. However, the underlying mechanism and therapeutic effect of ATL in the TNBC are largely unknown. In the present study, we found that ATL suppresses TNBC cell viability by reactive oxygen species (ROS) accumulation and subsequent ROS‐dependent endoplasmic reticulum (ER) stress both in vitro and in vivo. Thioredoxin reductase 1 (TrxR1) expression and activity of were significantly up‐regulated in the TNBC tissue specimens compare to the normal adjacent tissues. Further analyses showed that ATL inhibits the activity of TrxR1 both in vitro and in vivo in TNBC and knockdown of TrxR1 in TNBC cells sensitized ATL‐induced cell apoptosis and ROS increase. These results will provide pre‐clinical evidences that ATL could be a potential therapeutic agent against TNBC by promoting ROS‐ER stress‐mediated apoptosis through partly targeting TrxR1.

## 1 INTRODUCTION

Breast cancer is undoubtedly one of the most common malignancies and is the second leading cause of cancer‐related death for women worldwide.^1^ Triple‐negative breast cancer (TNBC) is characterized by the lack of oestrogen, progesterone and erb‐b2 receptor tyrosine kinase 2 receptors expression, and accounts for approximately 15% of all breast cancers. Triple‐negative breast cancer is one of the biologically aggressive subtype of breast cancers, which is correlated with the poor prognosis, distant metastases, recurrence and worse mortality rates despite systemic therapy.^2^, ^3^ So far, due to molecular characteristics of TNBC, no efficient targeted therapy is available and surgery, radiotherapy, chemotherapy are major choices of the systemic treatment.^4^, ^5^ However, these common systemic therapies accompany with high relapse rate and significantly affect patient's quality of life.^6^ For example, generally used chemotherapeutic drugs, such as taxol and doxorubicin, exhibit high dose‐limiting toxicity to tumour cells as well as normal cells, which limit their clinical usage.^7^, ^8^ Although several clinical trials targeting TNBC specific molecules have been conducted, including poly ADP-ribose polymerase (PARP) and epidermal growth factor receptor (EGFR), but no significant improvements were observed in patients with TNBC.^9^ Therefore, there is an urgent need to discover more favourable and effective anti‐cancer drugs that improve therapeutic effects and prognosis of patients with TNBC.

Using plants treatment of cancers has a long history and there are more than 3000 plant species have been reported.^10^ Alantolactone (ATL), main bioactive compounds that are presented in many medicinal plants such as *Inula helenium*, *Inula racemosa*, L. *Inula japonica*, *Aucklandia lappa* and *Radix inulae*. Various pharmacological actions of ATL have been found, including anti‐inflammatory, anti‐microbial and anti‐cancer activities with no significant toxicity.^11^ Over the past decade, ATL has been reported to inhibit cancer cell proliferation in liver cancer,^12^ lung squamous cell carcinoma (SCC),^13^ breast cancer,^14^ cervical cancer^15^ and colorectal cancer.^16^ One recent study has shown that ATL inhibits MDA‐MB‐231 cell growth by induction of reactive oxygen species (ROS) and ROS‐mediated apoptosis.^17^ However, detailed anti‐cancer mechanism of ATL against TNBC is unclear, especially its direct target and effect in vivo are unknown.

Thioredoxin (Trx)/thioredoxin reductase (TrxR) system has been reported to involve in oxidative stress‐induced apoptosis in cancer cells.^18^, ^19^ Increased thioredoxin levels contribute to the cancer cell growth and resistance to chemotherapy.^19^ Various anti‐cancer drugs have been reported to directly or indirectly inhibit TrxR to reverse malignant characteristics.^20^ Thioredoxin reductase 1 (TrxR1) is a major redox regulator in mammalian cells and has been reported to be overexpressed in many cancer cells, such as cervical, hepatoma, pancreatic, gastric, lung and breast cancers.^21^, ^22^, ^23^, ^24^, ^25^, ^26^ Therefore, TrxR1 has emerged as a promising biomarker and drugable target for cancer therapy. This raises the questions whether TrxR1 is also overexpressed in TNBC, and whether ATL targets TrxR system to generate ROS.

In this study, we have examined the therapeutic effect and the anti‐cancer mechanism of ATL on TNBC cells both in culture and in xenografts. Our study showed that ATL suppresses TNBC cell viability through inducing apoptosis and causing cell cycle arrest by ROS‐dependent endoplasmic reticulum (ER) stress pathway both in vitro and in vivo. In addition, TrxR1 expression and activity were significantly increased in TNBC specimens compared to the normal breast specimens. We also found that ATL can effectively inhibit TrxR1 in vitro and in vivo. The present study provides strong evidences that ATL can be a clinical candidate drug for the treatment of TNBC.

## 2 MATERIALS AND METHODS

See Supplementary file.

## 3 RESULTS

### 3.1 ATL effectively induces cell death in human TNBC cells

**Figure 1:**
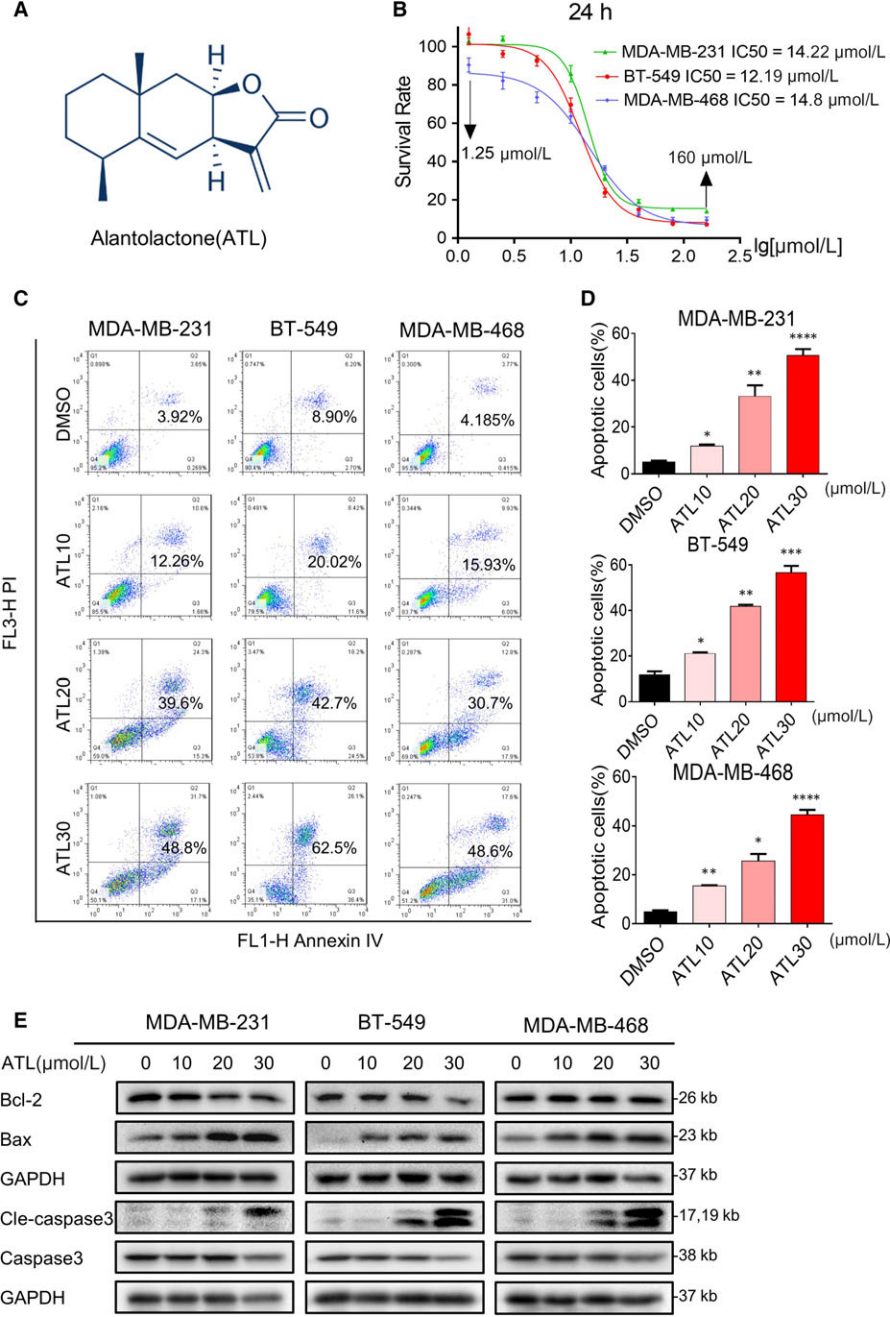
Alantolactone (ATL) induces cell apoptosis in triple‐negative breast cancer (TNBC) cells. A, Chemical structure of ATL. B, The effect of ATL on human TNBC cell viability. MDA‐MB‐231, BT‐549 and MDA‐MB‐468 cells were treated with increasing doses of ATL (1.25‐160 μmol/L) for 24 h. Cell viability was determined by MTT assay and the IC_50_ values were calculated. Data are expressed as Mean ± SEM (n = 3). C, Induction of apoptosis in TNBC cells was determined by annexin V/PI staining of cells following treatment with ATL (10, 20 or 30 μmol/L) for 24 h. D, Quantification of annexin V/PI staining showing the percentage of apoptotic cells following ATL treatment (*P < 0.05, **P < 0.01 and***P < 0.001 compared to Dimethyl sulfoxide (DMSO) control) （n = 3）. E, Western blot analysis of apoptosis‐associated proteins in cells challenged with ATL for 18 h. Glyceraldehyde 3‐phosphate dehydrogenase (GAPDH) was used as loading control

Previous studies have reported that ATL can suppress cell viability in several tumour types. Recent evidence suggests that ATL (Figure 1A) can inhibit human breast cancer cell line, MDA‐MB‐231 cell growth. To better understand the effect of ATL on TNBC cell viability, we treated three TNBC cell lines, MDA‐MB‐231， BT‐549 and MDA‐MB‐468, under increasing concentration of ATL (10, 20, 30 μmol/L) for 24 h. The cell viability was assessed by MTT assay. As shown in Figure 1B, ATL decreased the viability of MDA‐MB‐231, BT‐549 and MDA‐MB‐468 cells in a dose‐dependent manner, with IC_50_ values of 14.22, 12.15 and 14.8 μmol/L respectively.

We assessed whether ATL‐induced suppression of cells viability was associated with the increased apoptosis in cancer cells. Based on cell viability data, we selected concentration of 10, 20 and 30 μmol/L ATLs for the Annexin V/Propidium Iodide (PI) analyses. Our results showed that ATL induced significant dose‐dependent increased apoptotic death in TNBC cells (Figure 1C,D). Next, we characterized the nucleic morphological change by Hoechst staining. The control cells displayed normal, round nuclei with faint and even fluorescence, while the cells treated with ATL (30 μmol/L) exhibited highly and condensed fluorescent nuclei, a characteristic morphology of apoptotic cells (Figure S1A). To be more rigorous, we treated MDA‐MB‐231, BT‐549 and MDA‐MB‐468 cells with ATL and examined the levels of apoptosis‐related proteins by western blotting. Treatment of TNBC cells with ATL decreased Bcl‐2 (Figure 1E) while increased the levels of Bax. As caspase‐3 is a crucial component of the apoptotic machinery, and the cleavage of caspase‐3 is a central event in the process of apoptosis, we further examined the expression of cleaved caspase‐3. As shown in Figure 1E, the cleaved forms of caspase‐3 were increased in TNBC cells exposed to ATL. These data suggest that ATL could induce apoptosis of TNBC cells.

### 3.2 ATL effectively inhibits proliferation and colony formation of human TNBC cells

**Figure 2:**
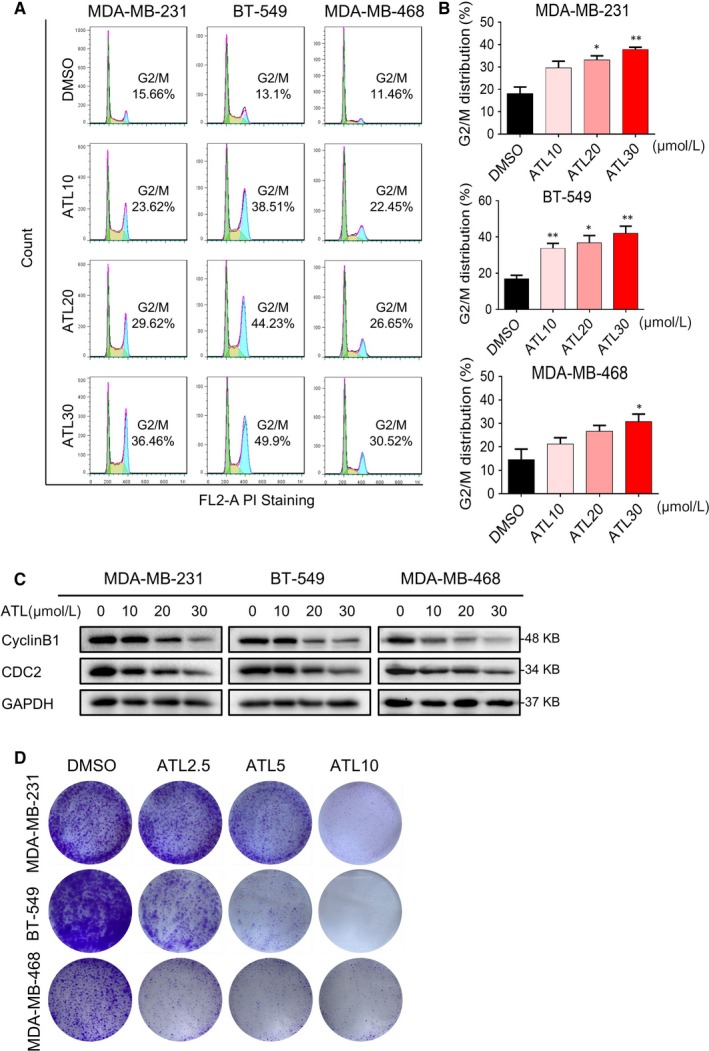
Alantolactone (ATL) inhibits proliferation, migration and colony formation in triple‐negative breast cancer (TNBC) cells. A, Cell cycle phase analysis following exposure of TNBC cells to ATL. Cells were exposed to ATL at 10, 20 or 30 μmol/L for 12 h. B, Flow cytometry histograms of cells stained with propidium iodide (PI). Quantification of flow cytometry data for G2/M phase arrest (*P < 0.05, **P < 0.01 compared to DMSO) (n = 3). C, Western blot analysis of G2/M cell cycle‐associated proteins in cells treated with ATL for 16 h. GAPDH was used as loading control. D, Effect of varying ATL concentrations on TNBC cells colony formation. Cells were incubated with ATL at 2.5, 5 or 10 μmol/L for 24 h and allowed to grow for 14 d (MDA‐MB‐231), 10 d (BT‐549) and 20 d (MDA‐MB‐468). Colonies were stained by crystal violet dye

We next assessed the effect of ATL on the TNBC cell growth. Propidium iodide staining of cells revealed remarkable accumulation of cells in the G2/M cell cycle phase in a concentration‐dependent manner, with a decrease in the population of cells in G1 and S phase, following exposure to ATL (Figure 2A,B). Western blotting analysis of G2/M phase‐related proteins also showed that obviously reduced expression levels of CyclinB1 and Cdc2 after treatment of cells with ATL (Figure 2C).

Furthermore, colony formation assay also showed that ATL significantly prevented colony formation of TNBC cells at 5 and 10 μmol/L levels (Figure 2D).

### 3.3 ATL increases ROS levels in human TNBC cells

**Figure 3:**
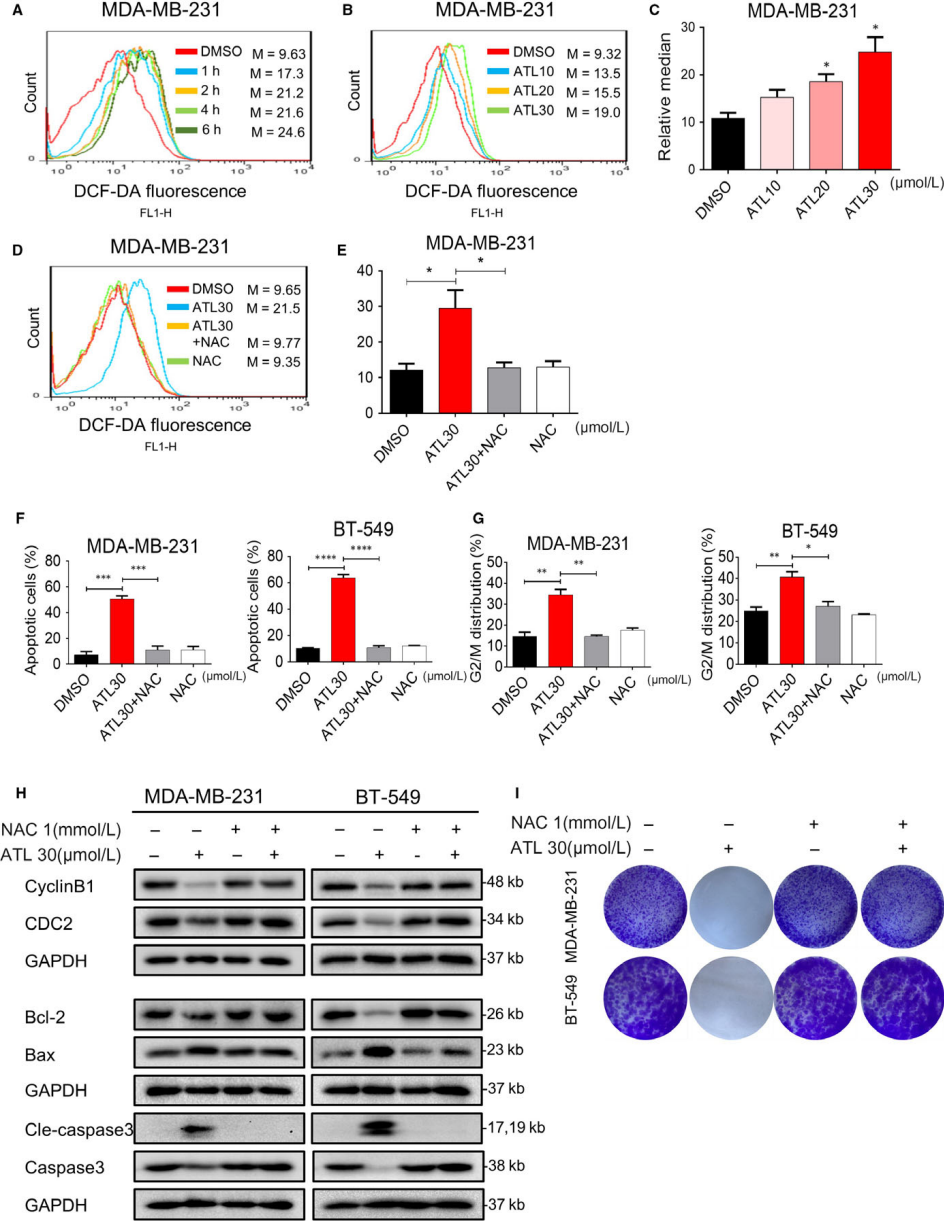
Alantolactone (ATL) increases ROS accumulation in human triple‐negative breast cancer (TNBC) cells. (A,B) Intracellular ROS generation was assessed by DCF fluorescence in MDA‐MB‐231 cells following exposure to ATL for different time periods (A, 30 μmol/L) and different concentrations (B). Quantification of data in (B) is shown in panel (C) [*P < 0.05 compared to DMSO control] (n = 3). D, MDA‐MB‐231 cells with or without 1 mmol/L NAC were exposed to ATL (30 μmol/L) for 1 h and intracellular ROS levels were measured by DCF fluorescence. Quantification of data in (D) is shown in panel (E) (*P < 0.05 compared to DMSO control) (n = 3). F, Apoptosis induction in cells exposed to ATL (24 h), with or without pre‐treatment with 1 mmol/L NAC for 1 h. Figure showing flow cytometry histograms. (***P < 0.001, ****P < 0.0001 compared to DMSO). Representative figures are shown in Figure S3B. G, G2/M phase accumulation in cells exposed to ATL (12 h), with or without pre‐treatment with 1 mmol/L NAC for 1 h. Figure showing flow cytometry histograms. (*P < 0.05, **P < 0.01 compared to DMSO) (n = 3). Representative figures are shown in Figure S3C and D. H, Western blot analysis of proteins in cells pre‐treated with 1 mmol/L NAC prior to 30 μmol/L ATL exposure. For cell cycle phase proteins, ATL exposure was carried out for 16 h and for apoptosis‐related protein, exposure was for 18 h. I, Colony formation in cells exposed to 30 mμmol/L ATL (24 h), with or without pre‐treatment with 1 mmol/L NAC for 1 h

Reactive oxygen species accumulation has been reported to induce apoptosis in several cancer cells.^27^, ^28^ To examine whether apoptosis and reduced viability in TNBC cells following ATL treatment are involved in increased ROS levels, we further treated cells using 2',7'‐Dichlorodihydrofluorescein diacetate (DCFH‐DA), which is rapidly oxidized by ROS to produce fluorescence 2',7'‐dichlorofluorescein (DCF) inside of cells. Treating MDA‐MB‐231 cells with ATL induced significant dose‐dependent increased fluorescence intensity compared with those of non‐treated cells (Figure 3A‐C). Next, we checked if ATL‐induced ROS is repressed by antioxidant. Pre‐treatment of MDA‐MB‐231 cells with N‐acetyl cysteine (ROS scavenger; N‐acetyl‐L‐cysteine (NAC), 1 mmol/L) significantly reduced ATL‐induced increases in DCF fluorescence as expected (Figure 3D,E).

We next wanted to check whether the negative growth signals from ATL can be attenuated by reduction of ROS levels. Pre‐treatment of cells with NAC (1 mmol/L) was able to attenuate ATL‐induced apoptosis (Figure 3F; Figure S1A,B) and cell cycle arrest (Figure 3G; Figure S1C). As expected, NAC prevented ALT‐induced alterations of apoptosis‐related proteins, Bcl‐2, Bax and cleaved caspase‐3; and G2/M‐phase proteins, cyclinB1 and CDC2 (Figure 3H). Furthermore, pre‐treatment of cells with NAC significantly attenuate the inhibition of colony formation (Figure 3I) caused by ATL. These finding suggests that the anti‐tumour effect of ATL, at least partly, depends on increasing ROS levels in human TNBC cells.

### 3.4 ROS‐dependent ER stress pathway contributes to ATL lethality in TNBC cells

**Figure 4:**
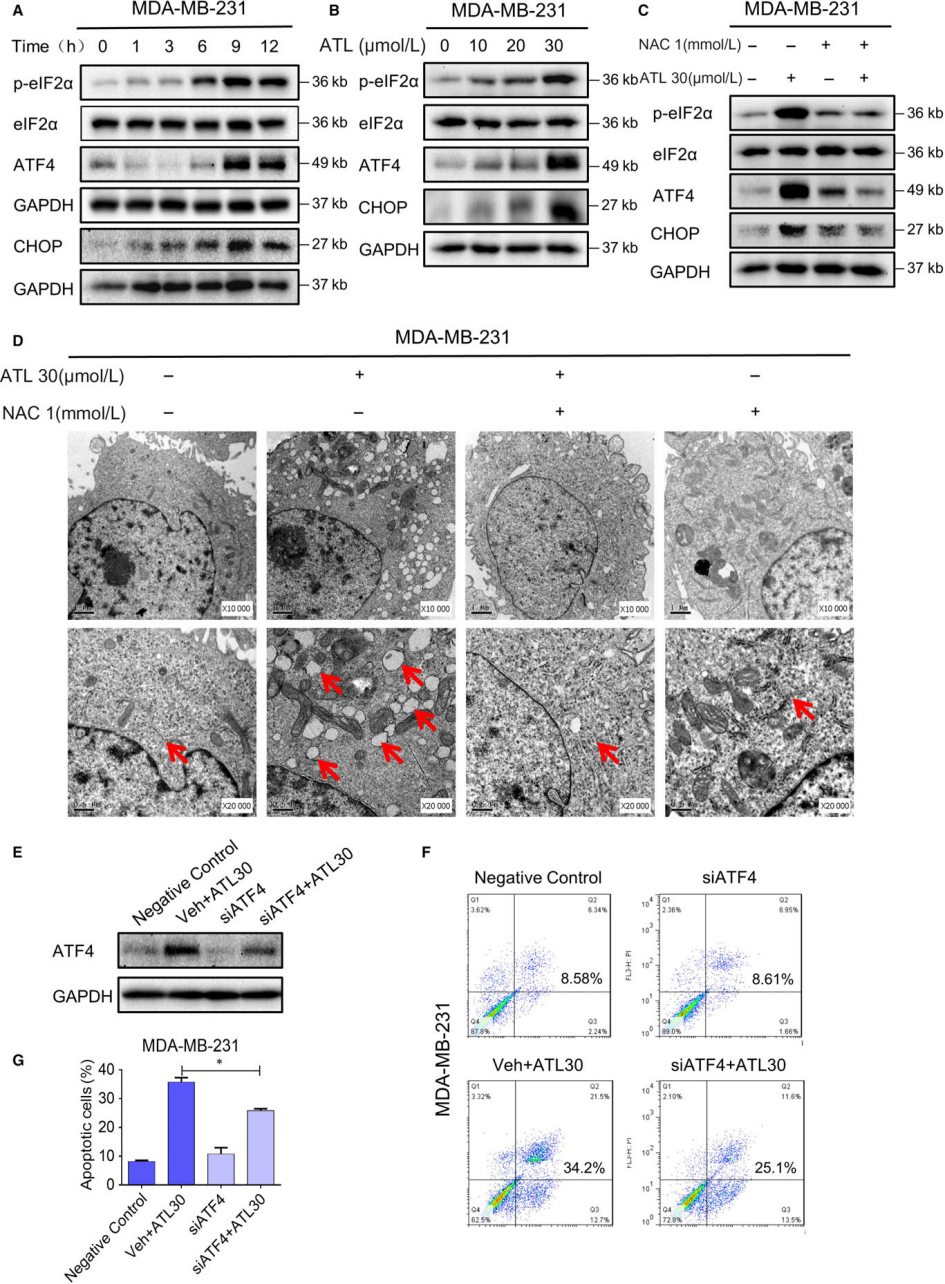
ROS‐dependent endoplasmic reticulum (ER) stress pathway is involved in alantolactone (ATL)‐induced triple‐negative breast cancer (TNBC) cell's growth inhibition. A, MDA‐MB‐231 cells were exposed to 30 μmol/L ATL for the indicated times. Protein levels of ATF4, phosphorylated eIF2α and CHOP were determined by western blot. GAPDH served controls. B, Western blot analysis of ER‐stress pathway associated proteins in MDA‐MB‐231 cells exposed to various doses of ATL for 9 h. C, MDA‐MB‐231 cells were pre‐treated with 1 mmol/L NAC prior to 30 μmol/L ATL exposure. Endoplasmic reticulum‐stress pathway associated proteins levels were detected by western blot. D, Electron microscopy images of MDA‐MB‐231 cells exposed to ATL (10 000× and 20 000× shown]. Cells were exposed to 30 μmol/L ATL for 9 h, with or without pre‐treatment with 1 mmol/L NAC for 1 h. **E**, MDA‐MB‐231 cells were transfected with ATF4 siRNA or control siRNA, ATF4 expression in MDA‐MB‐231 cells was determined by Western blotting after treating with ATL (30 μmol/L) for 9 h. **F**, Effect of ATF4 knockdown on ATL‐induced apoptosis was determined by annexin V/propidium iodide (PI) staining and flow cytometry. MDA‐MB‐231 cells were transfected with ATF4 siRNA or control siRNA for 24 h and then exposed to ATL (30 μmol/L) for 24 h. **G**, Quantification of annexin V/PI staining presented as the percentage of apoptotic cells following treatment (*P < 0.05, **P < 0.01 and***P < 0.001 compared to DMSO control) （n = 2）

Increased ROS levels and imbalance of the intracellular redox status increase unfolded proteins in the ER and induce ER stress response.^29^ Endoplasmic reticulum stress or unfolded protein response (UPR) induces protein kinase R (PKR)‐like ER kinase (PERK)‐mediated phosphorylation of eIF2a. Phosphorylated‐eIF2α allows preferential translation of activating transcription factor 4 (ATF4), while blocking cap‐dependent protein translation. In the ER stress pathway, ATF4 is a key transcription factor which mediates the induction of the pro‐death transcriptional regulator DNA damage inducible transcript 3 (CHOP). Thus, we tested the expressions of these ER stress‐related proteins in TNBC cells following ATL treatment, and examined whether alternation of these proteins related to the ATL‐mediated ROS generation. The time course result shows that ATL (30 μmol/L) could significantly induce the phosphorylation of eIF2α, protein levels of ATF4 and CHOP in MDA‐MB‐231 cells. And the expression levels of these ER stress‐related proteins reached the peak about 9 h following treatment (Figure 4A). We further noted that dose‐dependent activation of ER stress by ATL treatment in TNBC cells (Figure 4B; Figure S2A). Moreover, pre‐treatment of cells with NAC (1 mmol/L) markedly attenuated the induction of these ER stress related proteins (Figure 4C; Figure S2B). We next investigated the effect of ATL on the morphology of ER in MDA‐MB‐231 cells. Electron microscopy showed that a 9 h ATL challenge is conspicuous to cause ER swelling, while pre‐treatment with NAC totally reversed this morphological alteration (Figure 4D). Electron microscopy indeed revealed swollen mitochondria with disrupted cristae in cells exposed to ATL (Figure S3A). To determine ATL treatment indeed causes ER stress and UPR response in TNBC cells, we evaluated XBP1S and ATF6 expression in TNBC cells after treatment with ATL. The IRE1α‐XBP1S pathway has been reported to enhance or suppress cancer progression in different contexts.^30^, ^31^ On the other hand, ATF6α‐dependent UPR has shown cytoprotective functions leading to oncogenic roles in tumourigenesis.^32^, ^33^ We found that ATL treatment markedly increased XBP1S expression (Figure S2C), but decreased ATF6 expression in MDA‐MB‐231 cells (Figure S2D) indicating that ER stress and a UPR response are indeed activated after treatment with ATL. Next, knockdown analysis was performed to further investigate that sustained ER stress indeed contributes to the ALT‐induced cell death. Knockdown of ATF4 by siRNA markedly reduced ATF4 expression in MDA‐MB‐231 cells (Figure 4E), accordingly we observed that reduced apoptotic cells in ATF4 knockdown cells treated with ALT (Figure 4F,G). These findings indicate that ALT‐induced cell death is at least in part a result of sustained ER stress.

### 3.5 TRXR1 is up‐regulated in clinical TNBC cancer tissues and inactivated by ATL

**Figure 5:**
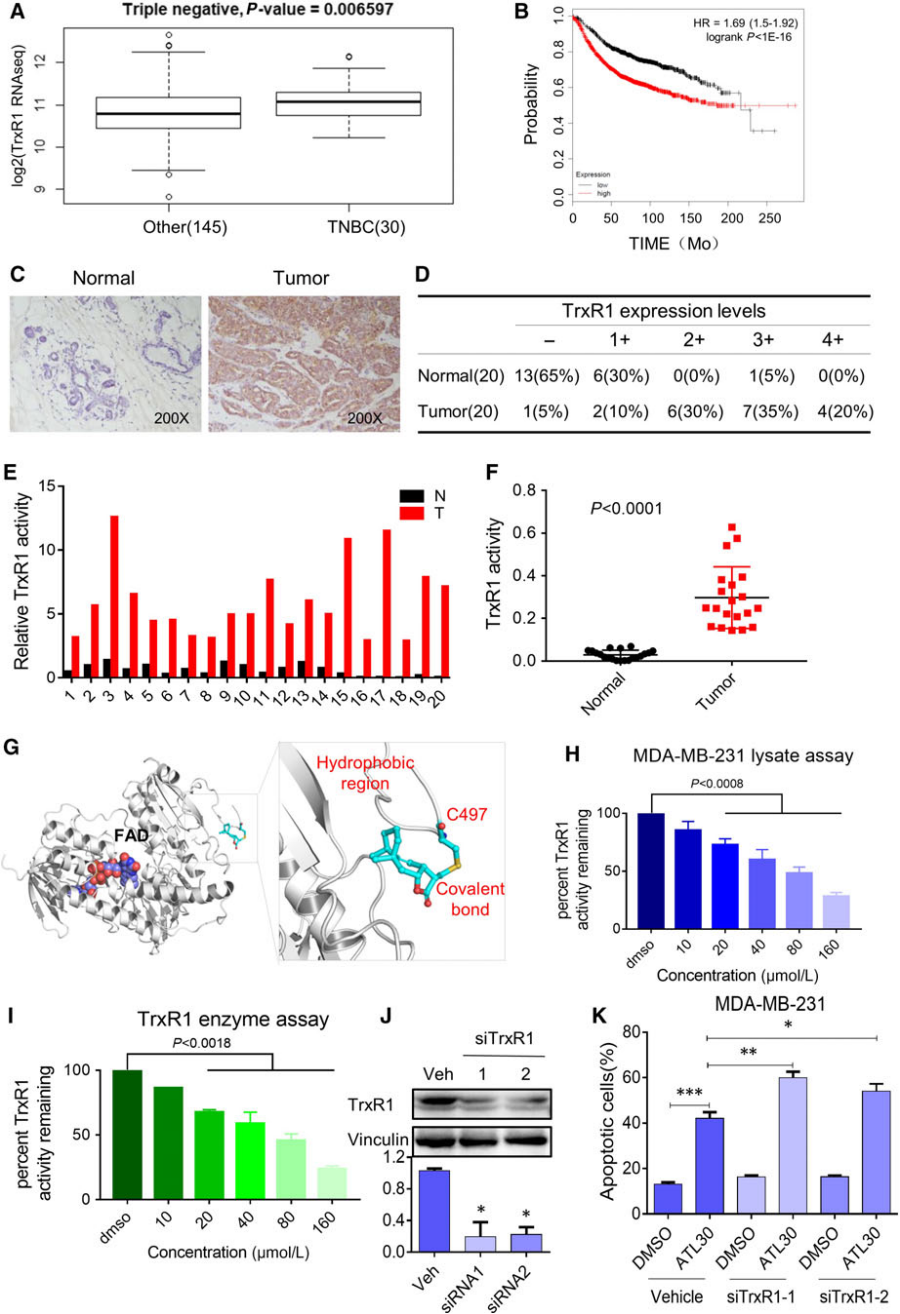
Alantolactone (ATL) inhibits Thioredoxin reductase 1 (TrxR1) activity in triple‐negative breast cancer (TNBC) (A) TrxR1 mRNA (GSE59590) levels in human TNBC tissues and other breast cancer tissues. The GSE59590 dataset includes 30 TNBC samples and 145 other forms of breast cancer samples. B, Kaplan‐Meier plots of overall survival of breast cancer patients, stratified by expression of TrxR1 (3021 patients). Data obtained from the Kaplan‐Meier plotter database (kmplot.com/analysis). C, Representative immunohistochemical staining for TrxR1 in TNBC tissues (T) and adjacent normal breast tissues (N) from the same patient. D, Summary of tissue immunohistochemical staining data for TrxR1 in 20 pairs of clinical TNBC tissues (T) and adjacent normal breast tissues (N). E, Endpoint insulin reduction assay confirmed relative TrxR1 activity in the TNBC tissues (T) and the paired adjacent normal breast tissues (N) from the same patients. F, Thioredoxin reductase 1 activity in human TNBC tissues and normal breast tissues (n = 20). G, Molecular docking of ATL with TrxR1 protein was simulated by docking software. (H,I) Thioredoxin reductase 1 enzyme activity was measured with different concentrations of ATL treatment in MDA‐MB‐231 lysates (H) and rhTrxR1 (I) by end‐point insulin reduction assay (n = 3). J, The TrxR1 expression was determined by Western blotting after knockdown with two different siRNAs for 48 h (n = 3). K, Knockdown of TrxR1 in MDA‐MB‐231 cells significantly promotes ATL induced apoptotic cells (n = 3)

Thioredoxin reductase 1 overexpression has been reported in several malignancies. Overexpression of TrxR1 was associated with aggressive tumour growth and poor survival. In addition, it has been reported that TrxR system contributes to tumour cell resistance to oxidative stress.^18^ We firstly examined TrxR1 mRNA expression in TNBC. GSE59590 dataset showed that TrxR1 mRNA expression was significantly up‐regulated in patients with TNBC compared to the patients without TNBC in breast cancer specimens (Figure 5A). The Kaplan‐Meier survival analysis using 3021 available breast cancer patients from the Kaplan‐Meier Plotter showed that high expression of *TrxR1* was significantly associated with poor prognosis of breast cancer patients (Figure 5B). We further collected 20 breast cancer specimens from the patients with TNBC and checked TrxR1 levels in biopsy specimens by using immunochemical staining (Figure 5C‐D). The clinical information of patients was listed in the Table S1. Our results showed that TNBC specimens displayed significantly increased TrxR1 immunoreactivity compared with the normal adjacent breast tissue from the same patient. Of note, 85% TNBC tissue specimens showed moderate to strong TrxR1 expression; however, only 5% normal adjacent tissues present moderate to strong TrxR1 expression. These results indicate that TrxR1 expression is significantly up‐regulated in TNBC tissues. Further, we tested the TrxR1 enzyme activity by using the 5,5‐dithio‐bis‐(2‐nitrobenzoic acid) (DTNB) assay. As shown in Figure 5E and F, the activities of TrxR1 in tumours were significantly up‐regulated compared to the corresponding normal breast specimens. All together, these findings indicate that TrxR1 might play pivotal functions in TNBC carcinogenesis.

We next want to know whether TrxR1 is a target of ATL in TNBC cells. A recent study showed that ATL inhibits the recombinant TrxR1 in HeLa cells.^34^ To investigate the structural mechanism of ATL binding to the TrxR1 protein, we performed a molecular simulation of ATL‐TrxR1 complex using AutoDock. Our result showed that ATL not only can insert into the C‐terminal active site of TrxR1 but also form a strong covalent bond (Figure 5G). It has been reported that the redox motif containing Cys‐497, Sec‐498 plays a vital role in enzyme inactivation, thus competitive inhibition to these residues could significantly desensitize the enzyme.^35^, ^36^ During the docking process, the alkenyl in ATL was detected as Michael acceptor to form a hard covalent bond with Cys‐497 while the cyclohexane part inserted into the hydrophobic pocket. Thus occupying the redox active centre may block the nature enzymatic recognition. This docking study suggests that TrxR1 is the potential target of ATL and blocking the critical residues in redox centre could inhibit its enzyme activity. We further tested the direct inhibitory effects of ATL on TrxR1 enzyme activity by using DTNB assay. When lysates prepared from MDA‐MB‐231 cells were incubated with various concentrations of ATL for 2 h, the DTNB reducing activity of TrxR1 decreased in a dose‐dependent manner (Figure 5H). We confirmed these results by measuring TrxR1 enzyme activity in pre‐reduced recombinant human TrxR1 (Figure 5I). We next examined the importance of TrxR1 in the cytotoxic effect of ATL. Knockdown of TrxR1 by two different TrxR1 siRNAs markedly reduced TrxR1 expression in MDA‐MB‐231 cells (Figure 5J). The TrxR1 knockdown by either siRNA1 or siRNA2 resulted in increased apoptosis and ROS levels in MDA‐MB‐231 cells induced by ATL (Figure 5K; Figure S4A,B). These findings indicate that ATL inactivates TrxR1, consequently increased cellular ROS in human TNBC cells and TrxR1 plays critical functions in ATL‐induced cell apoptosis in TNBC.

### 3.6 ATL inhibits the growth of MDA‐MB‐231 cell xenograft in vivo, accompanied with decreased TRXR1 activity and increased ROS level

**Figure 6:**
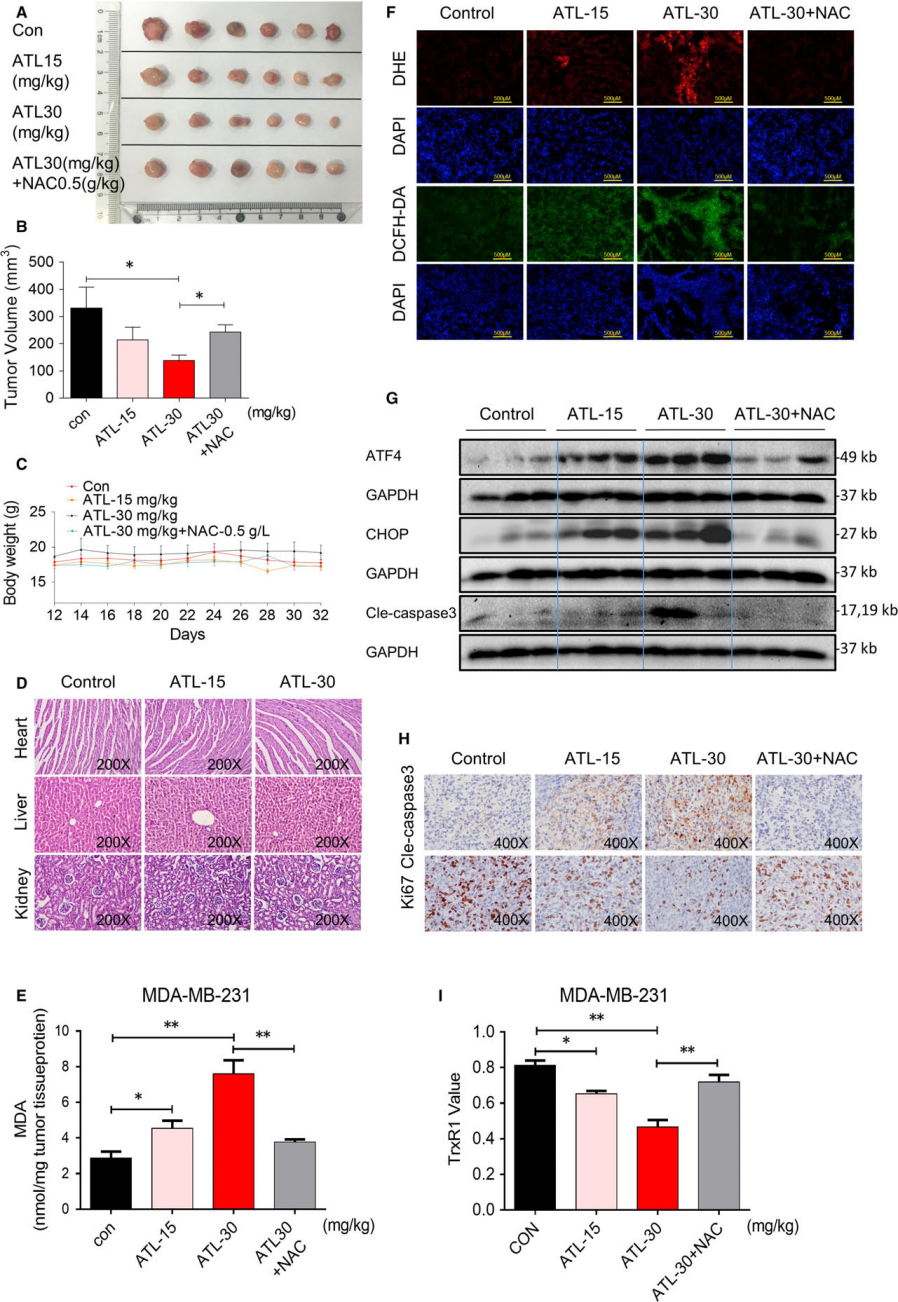
Alantolactone (ATL) inhibits MDA‐MB‐231 cell xenograft growth in vivo, accompanied with decreased Thioredoxin reductase 1 (TrxR1) activity and increased ROS level. (A,B) Tumour volume in vehicle and ATL with or without NAC treated mice. Injected MDA‐MB‐231 cells in the flanks of nude mice and tumours were allowed to develop for 12 d. Mice were then treated with ATL at 15 or 30 mg/kg interperitoneally for 20 d. NAC (0.5 g/L) was administered in the drinking water for one group mice with 30 mg/kg ATL for the same days. *P* < 0.01]. Images showing tumour tissues at day 32 (A) and the final tumour volume (B). Tumour volumes were calculated as described in the methods section. (*P < 0.05). C, Body weight of the mice in four groups (n = 6). D, Hematoxylin and eosin staining of heart, liver and kidney specimens (magnification 200×). E, Levels of malondialdehyde (MDA) in tumour tissue lysates (*P < 0.05, **P < 0.01 compared to vehicle treated group) (n = 3). F, Fluorescence images of tumour specimens stained with DHE (red) and DCFH‐DA (green) and tissue sections were counterstained with 4′,6‐diamidino‐2‐phenylindole (blue). Increased fluorescence intensity is indicative of increased ROS levels (magnification 200×). G, Western blot analysis of ATF4, CHOP and Cleaved‐caspase3 levels in tumour tissues. GAPDH was used as loading control. H, Representative immunohistochemical staining images of cell proliferation marker (Ki‐67) and apoptosis marker (Cleaved caspase‐3) in tumour tissues. I, Activity of TrxR1 in tumour tissue lysates as determined by end‐point insulin reduction assay. (*P < 0.05, **P < 0.01, compared to vehicle treated mice) (n = 3)

We found that ATL effectively induces apoptosis and inhibits proliferation in TNBC cells through ROS‐dependent ER‐stress pathway by decreasing TrxR1 activity. To determine the impact of ATL in vivo, we produced MDA‐MB‐231 xenografts in nude mice. We treated mice with ATL at 15 or 30 mg/kg for 20 days. NAC (0.5 g/L) was administered in the drinking water for a group of mice which are treated with 30 mg/kg ATL for the same days. The high dose (30 mg/kg) of ATL decreased tumour volume in mice, while the NAC‐treated group was able to diminish the ATL‐induced tumour growth inhibition (Figure 6A,B). Without significant changes in body weights were observed in ATL‐treated mice (Figure 6C). Haematoxylin and eosin staining analyses of vital organs (heart, liver and kidney) did not show any histological change that would indicate ATL toxic effects (Figure 6D). Furthermore, we observed that ATL treatment increased the levels of lipid peroxidation product malondialdehyde (MDA) in tumour tissues (Figure 6E) indicating increased ROS production. In addition, NAC significantly abolished the increasing MDA level induced by ATL (Figure 6E). We next stained the tumour tissues with ROS‐sensitive Dihydroethidium (DHE) and DCFH‐DA. DHE forms a red fluorescent product upon reaction with ROS and intercalates with DNA. Microscopic examination revealed that increased DHE and DCFH‐DA fluorescence signals in tumour tissues from mice with ATL treatment, indicating increased ROS levels in mice with ATL treatment (Figure 6F). NAC treatment reversed the ATL‐induced increase in ROS levels (Figure 6F). Western blot analyses of the tumour tissues showed that ATL treatment increased the levels of ATF4, CHOP (Figure 6G) indicating activation of the ER‐stress pathway in vivo. As expected, NAC prevented ALT‐induced alterations of ER‐stress related proteins (Figure 6G). We next assessed ATL‐induced apoptosis by cleaved caspase‐3 levels through both Western blot analyses and immunohistochemistry. We found that cleaved caspase‐3 levels were increased in tumour tissues following treatment with ATL (Figure 6G,H), while cell proliferation (ki‐67 levels) was decreased (Figure 6H). Importantly, NAC treatment reversed the ALT‐induced alterations of cleaved caspase 3 and ki‐67 levels (Figure 6G‐H). More importantly, ATL also inhibited TrxR1 activity in MDA‐MB‐231 xenografts (Figure 6I). These results are consistent with the in vitro studies and show that ATL inhibits tumour growth by suppressing TrxR1 activity, subsequently elevates ROS production and induces ER‐stress‐related apoptosis.

## 4 DISCUSSION

**Figure 7:**
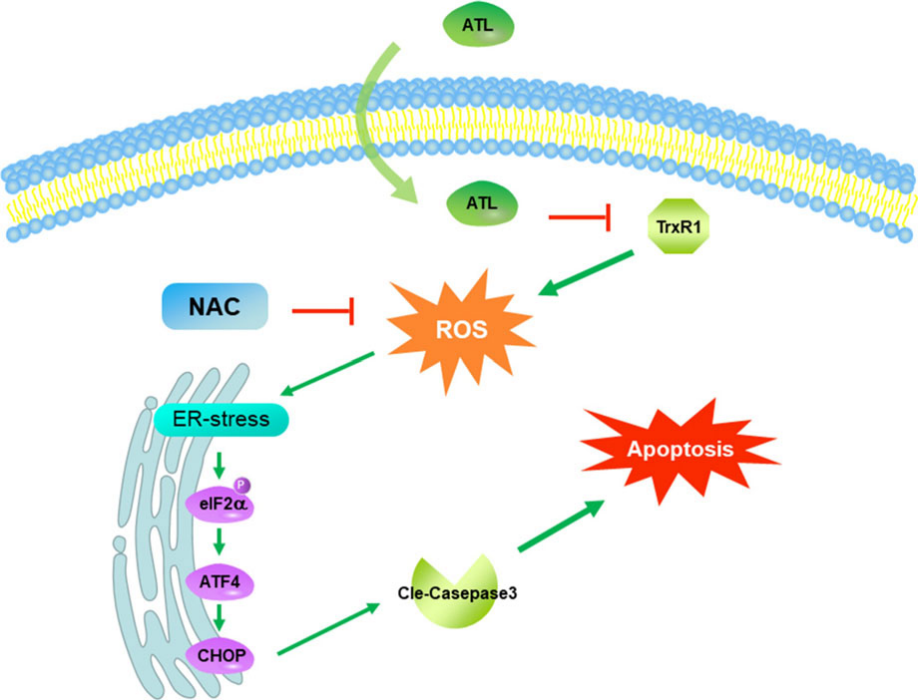
Schematic illustration of the proposed anti‐cancer mechanism of alantolactone (ATL) in triple‐negative breast cancer (TNBC). We show first time that ATL significantly inhibits thioredoxin reductase 1 (TrxR1) activity leading to increased ROS levels in the TNBC cells and its xenograft models. ROS accumulation causes the activation of the endoplasmic reticulum (ER) stress pathway/unfolded protein response and contributes the ROS‐induced cell death by activating caspases in TNBC cell lines and in a mouse pre‐clinical model

The development and investigation of natural products have always been one of the important means for human to search for anti‐cancer drugs. At present, most clinically approved drugs are derived from natural products or their analogues. Alantolactone, a sesquiterpene lactone extracted from medicinal plants, has been reported to have potential anti‐tumour activity against different types of cancers by promoting apoptosis. However, the underlying mechanisms of ATL and its target gene in TNBC are unclear. Here, we found that ATL significantly inhibits TrxR1 activity leading to increased ROS levels both in vitro and in vivo in TNBC. ROS accumulation causes the activation of the ER stress pathway/UPR and subsequent cell death by activating caspases in TNBC cell lines and in a mouse pre‐clinical model (Figure 7).

It has been reported that ATL induces apoptosis in HepG2 liver cancer cells by modulating the protein levels of Bcl‐2 family and activation of caspases, as well as loss of mitochondrial transmembrane potential and release of cytochrome c.^12^ Similar results were also observed in SK‐MES‐1 lung SCC cells.^15^ ATL also induces apoptosis of human cervical cancer cells via ROS generation, further study showed that ATL‐induced ROS overload triggers SW480 and SW1116 colorectal cancer cell death through induction of massive oxidative DNA damage and subsequent activation of the intrinsic apoptosis pathway.^15^, ^16^ It has also been reported that ATL exerts anti‐cancer activity via ROS‐mediated mitochondrial dysfunction in TNBC cell line, MDA‐MB‐231 cells and human colon cancer cell line, RKO cells.^17^, ^37^ However, it has not been examined that the target gene of ATL in TNBC and therapeutic effects of ATL in a mouse pre‐clinical model. We found that ATL could inhibit TNBC progression through causing apoptotic cell death, arresting of cell cycle and inhibiting colony formation by elevating ROS levels. Our study further demonstrated that ATL‐induced oxidative stress involved in ER stress and mitochondrial swelling. Additionally, we also found that ATL inhibits the growth of pre‐clinical human MDA‐MB‐231 xenograft model without cytotoxicity in major organs through targeting the same signalling pathway.

We have found that ATL promotes ER stress by ROS induction resulting in apoptotic cell death. To better understand how ATL influences this signalling pathway, we intend to find the target gene of ATL which involved in ROS‐ER stress‐mediated cell death. Recently, one research article has shown that ATL is an inhibitor of TrxR induces generation of ROS in HeLa cells.^34^ TRXR1 is a major redox regulator in mammalian cells and is widely expressed in numerous types of tissue cells. Higher levels of TrxR1 have been observed in various malignancies including breast cancer.^21^, ^22^, ^23^, ^24^, ^25^, ^26^, ^38^ In addition, it has been demonstrated that TrxR1 plays an important function in tumour growth, progression, metastasis and chemotherapy resistance.^39^, ^40^, ^41^

Our patient sample analyses, in agreement with publicly available GSE59590 dataset validated that TrxR1 expression and its activity were significantly increased in TNBC tissue samples compared to the NAT and other types of breast cancer samples. In addition, our study first time showed that ATL can suppress TrxR1 activity in the TNBC cell line MDA‐MB‐231 cells and its xenograft models. ATL inhibits TrxR1 activity, suppresses TNBC cell growth in xenografts by increasing accumulation of ROS in tissues, and leading to ROS‐dependent ER stress. Taken together, our study indicates that TrxR1 may serve as a promising biomarker and target molecule for TNBC therapy.

In conclusion, our study showed the potential usefulness and novel molecular mechanism of ATL in the treatment of TNBC. We found that ATL treatment resulted in significant ROS accumulation by inhibition of TrxR1 activity. ROS accumulation caused the activation of the ER stress‐mediated apoptotic pathway both in vitro and in vivo. Considering no efficient targeted therapy in TNBC and ATL effectively inhibits tumour growth of TNBC in vivo without obvious side effects, ATL might be a potential anti‐tumor drug in TNBC.

## CONFLICTS OF INTEREST

The authors declare no potential conflicts of interest.

## AUTHORS’ CONTRIBUTIONS

Ri Cui and Guang Liang conceived the idea and designed the research. Changtian Yin, Xuanxuan Dai, Xiangjie Huang, Qiulin Zhou, Chengguang Zhao and Peng Zou performed in vitro experiments. Changtian Yin, Xuanxuan Dai and Xiangjie Huang performed mice xenograft experiments. Changtian Yin, Wangyu Zhu, Xi Chen, Vinothkumar Rajamanickam and Ouchen Wang provided and analysed patient samples. Ri Cui, Xiaohua Zhang, Guang Liang, Xuanxuan Dai and Changtian Yin analysed the data. Ri Cui, Xiaohua Zhang and Changtian Yin wrote the manuscript.
