# Supplementary material for: Alantolactone promotes ER stress‐mediated apoptosis by inhibition of TrxR1 in triple‐negative breast cancer cell lines and in a mouse model
Source: J Cell Mol Med. 2019 Jan 4;23(3):2194–206. doi: 10.1111/jcmm.14139 (PMC6378194; doi:10.1111/jcmm.14139)
Supplement: Supplementary file 1 [file JCMM-23-2194-s001.doc]

***Supporting information***

**Alantolactone promotes ER stress mediated apoptosis by inhibition of TrxR1 in triple-negative breast cancer cell lines and in a mouse model**

Changtian Yin^1,2#^, Xuanxuan Dai^2#^, Xiangjie Huang^1^, Wangyu Zhu^3^, Xi Chen^1^, Qiulin Zhou^1^, Canwei Wang^4^, Chengguang Zhao^1^, Peng Zou^1^, Guang Liang^1^, Vinothkumar Rajamanickam^1^, Ouchen Wang^2^, Xiaohua Zhang^2*^ and Ri Cui^1*^

^1^ Chemical Biology Research Center, School of Pharmaceutical Sciences, Wenzhou Medical University, Wen zhou, Zhejiang, 325035, China

^2^ Department of Thyroid and Breast Surgery, The First Affiliated Hospital of Wenzhou Medical University, Wenzhou, Zhejiang, 325035, China

^3^ Cell and Molecular Biology Laboratory, Zhoushan Hospital of Wenzhou Medical University, Zhoushan, Zhejiang, 316021, China

^4^ Afﬁliated Yueqing Hospital and School of Pharmaceutical Sciences, Wenzhou Medical University, Wenzhou, Zhejiang, 325600, China

# These authors have contributed equally to this work

* Corresponding author:

Ri Cui, Chemical Biology Research Center, School of Pharmaceutical Sciences,

Wenzhou Medical University, Wenzhou 325035, China

E-mail: wzmucuiri@163.com

Phone number: +86-0577-86699396

Fax: +86-0577-86699396

* Co-corresponding author:

Xiaohua Zhang, Department of Thyroid and Breast Surgery,

The First Affiliated Hospital of Wenzhou Medical University,

Wenzhou, Zhejiang 325035, China

E-mail address: zhangxiaohua925@126.com

Tel: +86-0577-55579462

**Supplementary Materials and methods**

**Reagents**

Alantolactone (ATL) and the antioxidants NAC was obtained from Aladdin (Shanghai, China). Antibodies against Bcl-2 (sc-492), Bax (sc-493), Cdc2 (sc-54), TrxR1 (sc-28321), GAPDH (sc-32233), XBP-1S (sc-7160) and horseradish peroxidase-conjugated secondary antibodies were purchased from Santa Cruz Biotechnology (Santa Cruz, CA). Antibodies against cleaved caspase-3 (9664S), caspase-3 (9662S), cyclin B1 (4135S), ATF4 (11815S), p-eIF2α (3398S), eIF2α (9722S), CHOP (2895S) and ATF-6 (65880T) were purchased from Cell Signaling Technology (Danvers, MA). Antibodies against Ki67 (ab16667) was purchased from Abcam (Cambridge, UK). FITC Annexin V apoptosis Detection Kit I and Propidium Iodide (PI) were purchased from BD Pharmingen (Franklin Lakes, NJ). Reactive oxygen species probe DCFH-DA was purchased from Thermo Fisher (Carlsbad, CA, USA).

**Cells and cell culture**

Human TNBC cell lines (MDA-MB-231, BT-549 and MDA-MB-468) were purchased from the Institute of Biochemistry and Cell Biology, Chinese Academy of Sciences (Shanghai, China). MDA-MB-231 cells were cultured with DMEM high glocose medium (Gibco, Eggenstein, Germany), BT-549 were cultured with RPMI-1640 medium (Gibco, Eggenstein, Germany), whereas MDA-MB-468 were cultured with L-15 medium (Gibco, Eggenstein, Germany). All media formulations were supplemented with 10% heat-inactivated fetal bovine serum (Gibco, Eggenstein, Germany).

**Cell viability assay**

Cells were seeded at the density of 8 × 10^3^ per well in 96-well plates triplicate and allow to attach 24h in normal growth media. Initial studies were performed with 24 h cell culture condition with final concentrations of ATL at 1.25, 2.5, 5, 10, 20, 40, 80 and 160 μM. Following treatments, 25μL of fresh media containing 0.5 mg/mL MTT reagent was added to each well and cultured for an additional 4 h. DMSO (150 μL) was added to dissolve the formazan product and absorbance was measured at 490 nm using RT 6000 microplate reader. The percent inhibition rate was calculated as [1 − (treated/control)] × 100.

**Cell apoptosis and cell cycle analysis**

For apoptosis determination, MDA-MB-231, BT-549 and MDA-MB-468 cells were plated on 60-mm dishes for 24h, and treated with ATL (10, 20 or 30μM) for 24h, with or without 1mM NAC pretreatment for 1 h. Cells were then harvested, and evaluated for apoptotic cell death by double staining with FITC-conjugated Annexin V and Propidium Iodide (PI). For cell cycle analysis, TNBC cells were treated with ATL (10, 20 or 30μM) for 12h. Cells were then stained with PI at a ﬁnal concentration of 0.05mg/mL and incubated at 4°C for 20 min in the dark. Data were collected and analyzed using FACSCalibur flow cytometer.

**Western blot analysis**

Cells or tumor tissues were homogenized in protein lysis buffer, and debris was removed by centrifugation at 12000rmp for 10 min at 4 °C. Protein concentrations in all samples were determined by using Bradford protein assay (Bio-Rad, Hercules, CA). Protein samples were separated using 6–12% sodium dodecyl sulfate-polyacrylamide gels and transferred to PVDF membranes. The blots were blocked for 2h at room temperature with fresh 5% nonfat milk in TBST and then incubated with speciﬁc primary antibody in TBST overnight at 4°C. Horseradish peroxidase-conjugated secondary antibodies and ECL kit (Bio-Rad, Hercules, CA) were used for detection. Densitometric measurements were performed using Image J (National Institute of Health, MD).

**Colony formation assay**

To determine long-term effects, MDA-MB-231 and MDA-MB-468 cells were cultured at 2000 cells per wells and BT-549 were cultured at 1000 cells per wells in 60-mm dishes. The cells were then exposed to ATL (2.5, 5 or 10μM) for 24h. NAC pretreatments were carried out at 1mM for 1h. After being rinsed with fresh media, cells were allowed to grow for 10–20 days and stained with crystal violet solution (0.5 in 25%methanol) to assess colony growth.

**Determination of intracellular ROS**

Intracellular ROS contents were measured by ﬂow cytometry utilizing DCFH-DA. Brieﬂy, 5×10 ^5^ cells were plated in 60-mm culture dishes, allowed to attach overnight. Cells were then treated with ATL at concentrations(10, 20, or 30μM) and times(1, 2, 4, 6h) indicated. 1mM NAC pretreatment, where indicated, was carried out for 4h. Cells were stained with 10 μM DCFH-DA at 37°C for 30 min in the dark. DCF ﬂuorescence (produced in the presence of ROS) was analysed using ﬂow cytometry (FACSCalibur, BD Biosciences, CA). In all experiments, 8000 viable cells were analyzed.

**Hochest staining**

5×10^5^ thousand cells per well were plated in 6-well plates. After overnight incubation, the cells were treated with ATL (30μM) with or without 1mM NAC pretreatment for 1 h. After 24 h, a final concentration of 5 µg/mL Hoechst were added and incubated for 30 min at room temperature. The morphologic changes were observed with the fluorescence microscope (Nikon, Japan).

**Electron microscopy**

MDA-MB-231 cells were exposed to vehicle control (DMSO) or ATL (30 μM) for 9h. NAC pretreatment where applicable was carried out for 1h. Cells were ﬁxed in phosphate buffer (pH 7.4) containing 2.5% glutaraldehyde overnight at 4°C, post-ﬁxed in 1% OsO4 at room temperature for 60min, stained with 1% uranyl acetate, dehydrated through graded acetone solutions, and embedded in Epon. Areas containing cells were block-mounted and cut into 70nm sections and examined with the electron microscope (H-7500, Hitachi, Ibaraki, Japan).

**Patient samples**

This study was approved by the Institutional Research Human Ethical Committee of the Wenzhou Medical University for the use of clinical biopsy specimens and informed consent was obtained from the patients. A total of 20 TNBC patients biopsy samples were obtained. Clinical diagnosis was performed at the First Affiliated Hospital of Wenzhou Medical University during the period of 2017. TNBC tissues and matched tumor-adjacent morphologically normal breast tissues were frozen and stored in liquid nitrogen until further analyses. GSE59590 dataset was obtained from NCBI GEO Data Sets to analyze TrxR1 expression in the TNBC and the other forms of breast cancer.

**Determination of TrxR1 activity in cells or tumor tissues**

NADPH-reduced TrxR1 (170nM) was incubated with various concentrations of ATL for 2h at room temperature in 96-well plates. A mixture of TE buffer (50mM Tris-HCl, pH 7.5, 1mM EDTA, 50mL) containing 5,50-dithiobis (2-nitrobenzoate) (DTNB) and NADPH was added to achieve final concentrations of 2mM and 200mM, respectively. The linear increase in absorbance at 412nm during the initial 30 min was recorded. The same amounts of DMSO (1%, v/v) were added to the control experiments and the activity was expressed as the percentage of the control. The total protein content was determined by using the Bradford assay. TrxR1 activity in cell lysates or tumor tissues was measured by the end-point insulin reduction assay. Brieﬂy, 100μg total proteins were incubated in a final reaction volume of 50μL containing 100mM Tris-HCl (pH7.6), 0.3mM insulin, 660μM NADPH, 3mM EDTA, and 15μM E.coliTrx (Sigma, St.Louis, MO) for 30 min at 37°C. The reaction was terminated by adding 200μL of 1mM DTNB in 6M guanidine hydrochloride (pH 8.0). A blank sample, containing everything except Trx, was treated in the same manner. TrxR1 activity was determined at room temperature using SpectraMax M5 microplate reader (Molecular Devices, USA). The absorbance at 412nm was measured, and the blank value was subtracted from the corresponding absorbance value of the sample. The activity was expressed as the percentage of the control.

**Docking of ATL to the TrxR1 structural model**

CovalentDock, which was written based on Autodock, was implemented to predict the interaction of ATL binding to TrxR1[1]. The crystal structure of human TrxR1 used for this docking job was obtained from the Protein Data Bank (PDB ID 2ZZ0, chain A) and prepared by using PyMOL, including removing water molecules and adding hydrogens. Then minimization was performed to avoid local collision. A grid box of 60×60×60 points centering on the coordinate of -29.11, -1.26, and -6.55 was implemented, which enclose the whole redox motif. Other parameters were set as default during the docking.

**Cell transfections for gene silencing**

The two distinct sequences for TrxR1 siRNA oligonucleotides used in this study were synthesized by GenePharma (Shanghai, China). Sequence 1: (Sense: 5’-(GCAAGACUCUCGAAAUUAU)dTdT-3’,antisense:5’-(AUAAUUUCGAGAGUCUUGC)dTdT-3’). Sequence 2: sense5′-(CUUUGCAGCUGCGCUCAAA)dTdT-3′, antisense 5′-(UUUGAGCGCAGCUGCAAAG)dTdT-3′. The sequence for ATF4 siRNA is sense: 5’-GCCUAGGUCUCUUAGAUGATT-3’, antisense: 5’-UCAUCUAAGAGACCUAGGCTT-3’. Half million MDA-MB-231 cells were plated in 60-mm dishes with 2ml medium. After 24h, siRNA against TrxR1, ATF4 or non-targeting control were transfected into the cells with the ﬁnal concentration of 50 nM using lipofectamine 3000 reagent (Invitrogen, Carlsbad, CA, USA). After 48h post-transduction, the cells were washed with complete media and plated with or without ATL for 24 hours for assessing apoptosis or ROS levels.

**In vivo xenografts**

Animal studies were performed in compliance with the ARRIVE guidelines[2, 3]. All animal experimental procedures complied with the Wenzhou Medical University’s Policy on the Care and Use of Laboratory Animals. Four-week-old, athymic BALB/c nu/nu female mice (16-19g, totally n=24) were purchased from Vital River Laboratories (Beijing, China). Mice were housed at a constant room temperature with a 12 h:12 h light/dark cycle and fed a standard rodent diet and water. The mice were randomly divided into four experimental groups. MDA-MB-231 cells were injected subcutaneously (1×10^7^ cells in 100μL of PBS) into the right flank of mice. Mice were treated by intraperitoneal (i.p.) injections of ATL at the dose of 15 or 30mg/kg body weight once every other day for 20 days. Control mice were received vehicle only. NAC (0.5g/liter) was administered in the drinking water for one group of mice that treated with 30mg/kg ATL for the same days. The tumor volumes were determined by measuring length (L) and width (W) and calculating volume (V = 0.5 × L × W^2^) at the indicated time points. At the end of experiment, the animals were sacrificed and the tumors were harvested for use in proteins expression and histology studies.

**Malondialdehyde (MDA) assay**

Tumors samples from nude mice were homogenized and sonicated in RIPA buffer on ice. Tissue lysates were centrifuged at 12,000 xg for 10 min at 4 °C to collect the supernatant. Total protein content was determined by the Bradford assay. MDA levels were measured by Lipid Peroxidation MDA assay kit (Beyotime Institute of Biotechnology). MDA levels were detected using multimode microplate readers (SpectraMax M5, Molecular Devices, USA) at 532nm.

**Immunohistochemistry and histology**

The harvested tumour tissues were fixed in 10% formalin, processed and embedded in parafﬁn. 5-micrometre-thick sections were placed on positively charged slides. Tissue sections were stained using routine immunohistochemical techniques and incubated with primarily antibodies against TrxR1 (1:50), cleaved caspase 3 (1:100) or Ki-67 (1:100) overnight. Conjugated secondary antibodies and diaminobenzidine (DAB) were used for detection. According to the percentage of positive cells in the mean average of five fields, immunohistochemical reactivity for TrxR1 was scored as follows: 0–5% (−), 5%–25 (1+), 25%–50% (2+), 50%–75% (3+), 75%–100% (4+). The TrxR1 expression graded as 3+ and 4+ were defined as strong, 2+ was defined as moderate and 1+ was defined as weak, respectively. Heart, liver and kidney were also stained with H&E for histological analysis and assessment of potential toxicity.

**ROS determination in tumor tissues**

Tumor tissue sections were stained with 10 μM DHE and 50 μM DCFH-DA for 30 min. Images were captured under a fluorescence microscope (Nikon, Japan).

**Statistical analysis**

All experiments were assayed in triplicate (n = 3). Data are expressed as means±SEM. All statistical analyses were performed using GraphPad Pro. Prism 7.0 (GraphPad, SanDiego, CA). All results were analysed using unpaired t-test for comparison between two groups. Values of at least P < 0.05 were considered statistically significant.

**References**

1. Ouyang X, Zhou S, Su CT, Ge Z, Li R, Kwoh CK. CovalentDock: automated covalent docking with parameterized covalent linkage energy estimation and molecular geometry constraints. J comput chem. 2013;34(4):326-36.

2. McGrath JC, Drummond GB, McLachlan EM, Kilkenny C, Wainwright CL. Guidelines for reporting experiments involving animals: the ARRIVE guidelines. Brit j pharmacol. 2010;160(7):1573-6.

3. McGrath JC, Lilley E. Implementing guidelines on reporting research using animals (ARRIVE etc.): new requirements for publication in BJP. Brit j pharmacol. 2015;172(13):3189-93.

**Supplementary Figure 1**


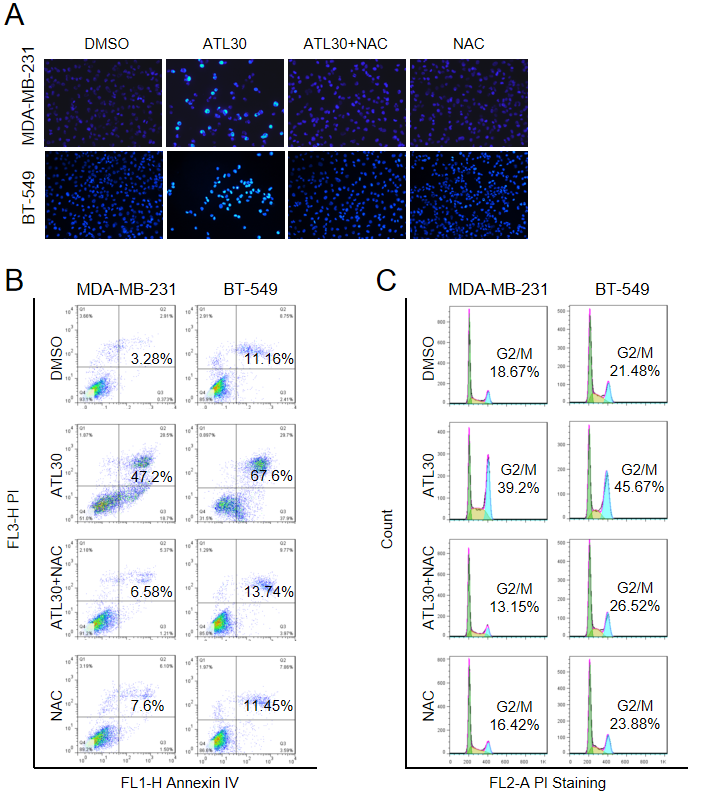


**Supplementary Figure 1. ATL induced apoptosis, growth arrest and inhibition of migration are dependent on ROS generation.**

(**A**) Hoechst staining showed typical apoptotic morphology changes of the nuclei after ATL treatment. TNBC cells were pretreated with or without NAC for 1h at the concentration of 1 mM, subsequently incubated with ATL (30μM) for 24 h, and the nuclei were stained by Hoechst. The representative fluorescence pictures were acquired by inverted fluorescence microscope. (**B**) Induction of apoptosis in TNBC cells was determined by annexin V/PI staining. The cells were exposed to ATL (30μM) for 24 h, with or without NAC pretreatment (1 Mm, 1 h). (**C**) The cell cycle was analyzed by Flow cytometric analysis in cells exposed with 30 µM ATL for 12 h. NAC pretreatment was performed at 1 mM for 1 h.

**Supplementary Figure 2**


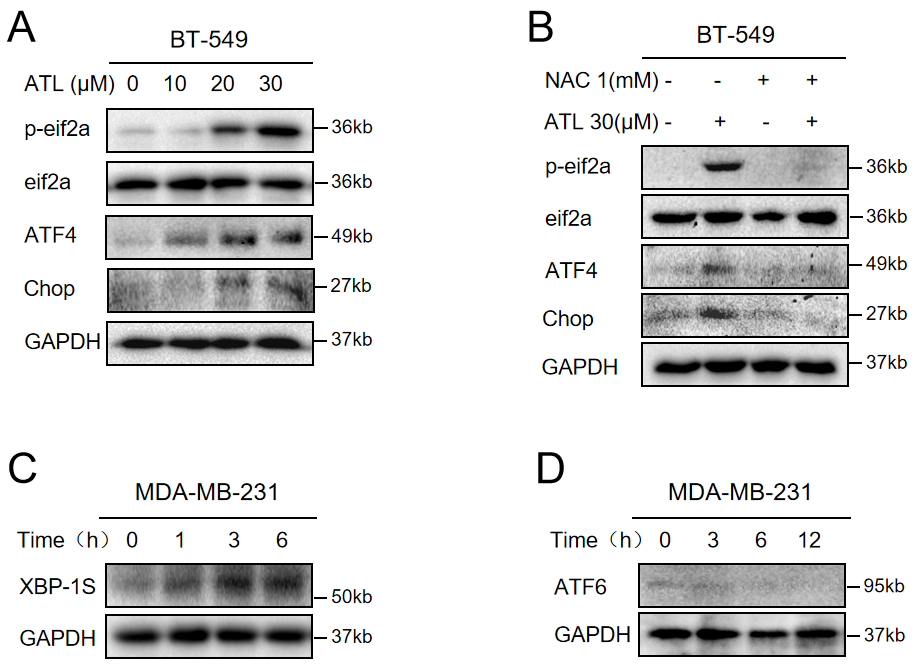


**Supplementary Figure 2. ATL induce ER stress in BT-549 cells.** (**A**) Western blot analysis of ER stress pathway related proteins in BT-549 cells treated with varying concentrations of ATL for 9 h (ATF4, p-eIF2α and CHOP). (**B**) NAC pretreatment was carried out at 1 mM for 1 h before ATL treatment (30μM). For panels A and B, GAPDH served as internal controls. (**C**) MDA-MB-231 cells were exposed to 30μM ATL for the indicated times. Protein levels of XBP-1S was determined by western blot. (**D**) Western blot analysis of ATF-6 in MDA-MB-231 cells treated with ATL(30μM) for the indicated times.

**Supplementary Figure 3**

**
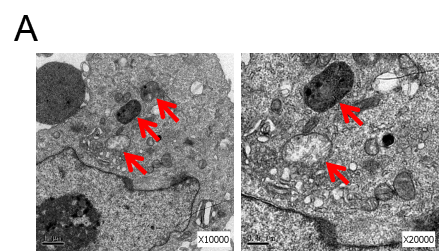
**

**Supplementary Figure 3. Morphological alterations in mitochondria following exposure of cells to ATL.**

(**A**) Electron microscopy images showing the effect of ATL on mitochondria. MDA-MB-231 cells were exposed to 30 µM ATL for 9h. NAC (1mM) pretreatment was carried out for 1 h. Images showing 10,000 and 20,000x mag. Red arrow indicates swollen mitochondria with disrupted cristae in cells exposed to ATL.

**Supplementary Figure 4**


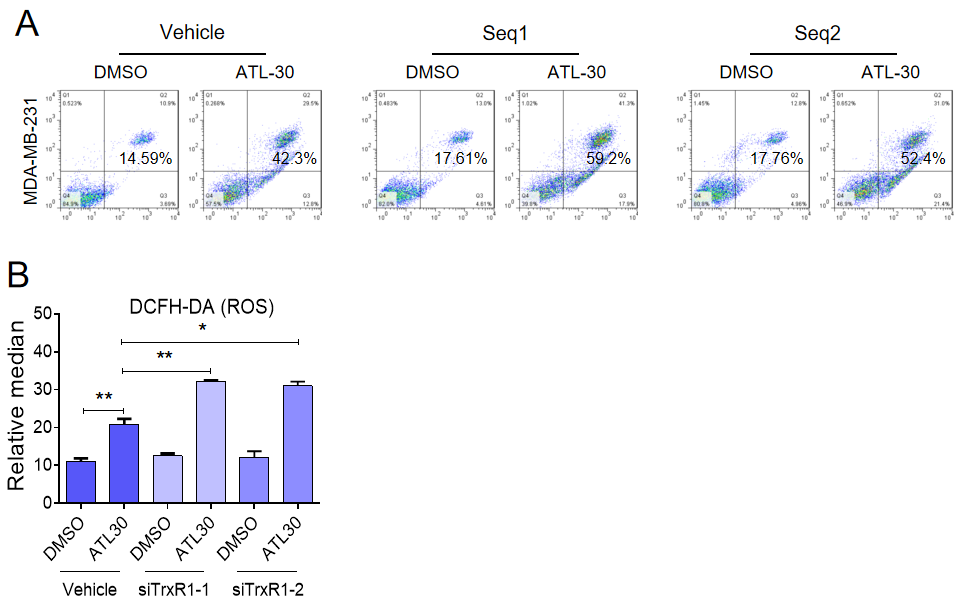


**Supplementary Figure 4. Knockdown of TrxR1 in MDA-MB-231 cells promotes ATL induced apoptotic cells and ROS levels.**

(**A**) Assessment of cell apoptosis in MDA-MB-231 cells following knockdown of TrxR1 and 30μM ATL treatment for 24h. (B) Knockdown of TrxR1 in MDA-MB-231 cells significantly promotes ATL induced ROS levels (n=3).

**Supplementary Table 1**

| Number | Sex | Age | Tumor size (cm) | Deep of invasion | Lymph node metastasis | Organ metastasis | Differentiation status | TNM Stage |
| --- | --- | --- | --- | --- | --- | --- | --- | --- |
| 1 | Female | 41 | 4.0 | T2 | N2 | M0 | Poor differentiation | ⅢA |
| 2 | Female | 61 | 3.0 | T2 | N0 | M0 | Poor differentiation | ⅡA |
| 3 | Female | 51 | 4.0 | T2 | N0 | M0 | Poor differentiation | ⅡA |
| 4 | Female | 52 | 1.0 | T1c | N0 | M0 | Moderate differentiation | Ⅰ |
| 5 | Female | 46 | 3.5 | T2 | N0 | M0 | Poor differentiation | ⅡA |
| 6 | Female | 64 | 4.0 | T2 | N0 | M0 | Poor differentiation | ⅡA |
| 7 | Female | 52 | 2.5 | T2 | N0 | M0 | Poor differentiation | ⅡA |
| 8 | Female | 55 | 2.5 | T2 | N1 | M0 | Poor differentiation | ⅡB |
| 9 | Female | 51 | 4.0 | T2 | N0 | M0 | Poor differentiation | ⅡA |
| 10 | Female | 52 | 3.0 | T2 | N0 | M0 | Poor differentiation | ⅡA |
| 11 | Female | 64 | 2.5 | T2 | N2a | M0 | Moderate differentiation | ⅢA |
| 12 | Female | 68 | 1.5 | T1c | N0 | M0 | unknown | Ⅰ |
| 13 | Female | 52 | 2.5 | T2 | N0 | M0 | Moderate differentiation | ⅡA |
| 14 | Female | 58 | 1.0 | T1c | N1 | M0 | Moderate differentiation | ⅡA |
| 15 | Female | 67 | 2.4 | T2 | N0 | M0 | Poor differentiation | ⅡA |
| 16 | Female | 68 | 1.5 | T1c | N0 | M0 | unknown | Ⅰ |
| 17 | Female | 60 | 3.0 | T2 | N1 | M0 | Poor differentiation | ⅡB |
| 18 | Female | 67 | 2.5 | T2 | N0 | M0 | Poor differentiation | ⅡA |
| 19 | Female | 64 | 2.5 | T2 | N2a | M0 | Moderate differentiation | ⅢA |
| 20 | Female | 60 | 3.0 | T2 | N1 | M0 | unknown | ⅡB |

## Supplementary Table 1. The Clinical Characteristics of Patients

The chart is based on TNM staging of UICC (International Union Against Cancer) eighth edition of Malignant Tumors.
